# ﻿*Breyniahiemalis* (Phyllanthaceae, Phyllantheae), a new species from Yunnan, south-west China

**DOI:** 10.3897/phytokeys.206.85241

**Published:** 2022-08-26

**Authors:** Feng Yang, Chao Chen, Jing-Yi Ye, Jian-Yong Wu, Huan-Chong Wang

**Affiliations:** 1 School of Life Sciences, Yunnan University, Kunming 650091, Yunnan, China; 2 Key Laboratory of Tropical Forest Ecology, Xishuangbanna Tropical Botanical Garden, Chinese Academy of Sciences, Mengla 666303, Yunnan, China; 3 Yuanjiang Savanna Ecosystem Research Station, Xishuangbanna Tropical Botanical Garden, Chinese Academy of Sciences, Yuanjiang 653300, Yunnan, China; 4 Yuxi Forestry and Grassland Bureau, Yuxi 653100, Yunnan, China; 5 School of Ecology and Environmental Science, Yunnan University, Kunming 650091, China; 6 Herbarium of Yunnan University, Kunming 650091, Yunnan, China

**Keywords:** *Breynia* sect. *Cryptogynium*, endemism, *Sauropus*, savanna, Yuanjiang River

## Abstract

*Breyniahiemalis* Huan C. Wang & Feng Yang (Phyllanthaceae), of sect. Cryptogynium (Müll.Arg.) Welzen & Pruesapan in subg. Breynia, is described from Yunnan, south-west China. It is known from only a single locality in the valley of the Yuanjiang River, and usually occurs in the understory of the savanna vegetation. It is characterized by its broadly elliptic to orbicular leaf blades, shallowly plate-like calyces of the staminate flowers, ovaries with clearly erose rim and urceolate capsules. Morphological comparisons with similar species are also presented.

## ﻿Introduction

The family Phyllanthaceae, a segregate from Euphorbiaceae*sensu lato*, is a pantropical group of herbs, shrubs and treelets (Kathriarachchi et al. 2005; Hoffmann et al. 2006). It currently consists of about 2000 species, with more than 1200 placed in the largest tribe Phyllantheae (Govaerts et al. 2000; Hoffmann et al. 2006). Within the Phyllantheae, the generic classification is still contentious (Hoffmann et al. 2006; Wagner and Lorence 2011; Pruesapan et al. 2012; Van Welzen et al. 2014; Jangid and Gupta 2016; Bouman et al. 2022). Recent molecular studies clearly demonstrated that *Phyllanthus* L. (in the traditional sense), the largest genus of Phyllanthaceae, with more than 800 species, is paraphyletic since *Glochidion* J. R. Forst. & G. Forst., *Breynia* J. R. Forst. & G. Forst. and *Synostemon* F. Muell. are nested within it (Bouman et al. 2021, 2022). Some authors suggested placing most species of the Phyllantheae in *Phyllanthus*. This would make *Phyllanthus* a large and morphologically heterogeneous group with more than 1200 species (e.g. Hoffmann et al. 2006; Wagner and Lorence 2011; Ralimanana et al. 2013; Kato and Kawakita 2017; Falcón-Hidalgo et al. 2020). Conversely, other authors (for example, Pruesapan et al. 2008; Pruesapan 2010; Van Welzen and Pruesapan 2010; Pruesapan et al. 2012; Bouman et al. 2018, 2021) prefer to divide *Phyllanthuss. l.* into several smaller, monophyletic genera that can be characterized morphologically. More recently, Bouman et al. (2022) split *Phyllanthuss. l.* into thirteen monophyletic and morphologically recognizable genera, where *Breynia* was kept as a separate genus.

*Breynia* and *Sauropus* Blume in the strict sense are closely related; both share bifid or emarginate styles, non-apiculate anthers, smooth seeds, and they generally possess sepal scales (Van Welzen et al. 2014). From the results of a well-sampled phylogenetic analysis, Van Welzen et al. (2014) expanded *Breynia* to include the south-east Asian species of *Sauropus* and reinstated the Australian genus *Synostemon*, previously relegated to a section of *Sauropus*, making each genus monophyletic and morphologically definable. The newly circumscribed *Breynia* is a moderately-sized genus of 85 to 90 species (Van Welzen et al. 2014; Bouman et al. 2022). It contains three infrageneric groups, including subg. Breynia and subg. Sauropus (Blume) Welzen & Pruesapan, with subg. Breyniasubdivided intosect.Breynia and sect. Cryptogynium (Müll. Arg.) Welzen & Pruesapan. Members of *Breynia* are distributed mainly from Australia to tropical and subtropical Asia, with a center of diversity in south-east Asia (Van Welzen 2003; Bouman et al. 2022; POWO 2022). Twenty species of *Breynia* are found in China, including 15 species formerly placed in *Sauropus*, and most species occur in the southern and south-western regions (Li 1994; Li and Esser 2008; Li and Gilbert 2008). Recently, a new species of *Breynia* was described from Yunnan, south-west China by Yang et al. (2022). This brings the total number of Chinese species to 21, of which five species belong to sect. Cryptogynium, seven species belong to sect. Breynia, and nine species belong to subg. Sauropus.

During fieldwork in Yuanjiang National Natural Reserve, Yunnan Province (SW China), in December 2015 and January 2022, we came across a dwarf plant with discoid staminate flowers and 3 stigmas spreading horizontally from the top of obconical ovary, obviously belonging to *Breynia* according to the generic delimitation of Van Welzen et al. (2014) and Bouman et al. (2022). This could not be assigned to any previously known species from China (Li and Gilbert 2008), or from any of the adjacent south-east Asian countries, i.e. Vietnam, Laos, Thailand and Myanmar (Beille 1927; Pham 2003; Van Welzen 2003; Chakrabarty and Balakrishnan 2018; Van Welzen 2022; Van Welzen and Chayamarit 2022). Comparison with morphologically similar species supports recognizing this plant as an undescribed species.

## ﻿Materials and methods

The new species was studied both in the field and in the herbarium. The collections of similar species housed at KUN, PE, XTBG, YUKU, and digital images available at the JSTOR Global Plants (https://plants.jstor.org/), the Chinese Virtual Herbarium (http://www.cvh.ac.cn/), and the Global Biodiversity Information Facility (https://www.gbif.org/) were reviewed. Surveys of pertinent taxonomic literature (for example, Beille 1927; Li 1994; Pham 2003; Van Welzen 2003; Ma et al. 2006; Li and Gilbert 2008; Van Welzen et al. 2014; Chakrabarty and Balakrishnan 2018; Van Welzen 2022; Van Welzen and Chayamarit 2022) were carried out. Measurements were made under a stereomicroscope (Olympus SZX2, Tokyo, Japan) using a ruler and a metric vernier caliper.

## ﻿Taxonomy

### ﻿ Breynia hiemalis

Taxon classificationPlantaeMalpighialesPhyllanthaceae

Huan C. Wang & Feng Yang sp. nov.

931AEDC0-7240-56BE-BE43-BB595B220C46

urn:lsid:ipni.org:names:77303992-1

Figs 1 , 2 , 3

#### Type.

China. Yunnan Province: Yuanjiang County, Pupiao, 600–700 m, 23°28'37"N, 102°10'37"E, in savanna on a mountain slope, 12 Jan. 2022, *H. C. Wang et al. YJ16225* (holotype YUKU-02074690!; isotypes YUKU!, PE!, HITBC!).

**Figure 1.**
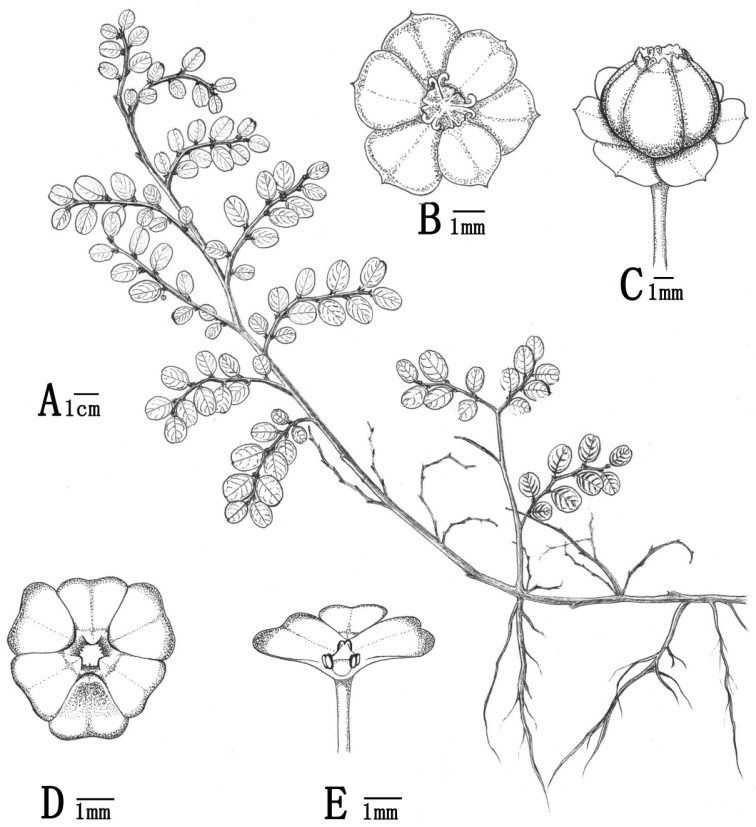
*Breyniahiemalis*. (Drawn by Jing-Yi Ye from type specimen *H. C. Wang et al. YJ16225*) **A** habit **B** pistillate flower (apical view) **C** fruit **D** staminate flower (apical view) **E** staminate flower (lateral view).

#### Diagnosis.

*Breyniahiemalis* can easily be distinguished from all morphologically similar species by plants glabrous throughout, by its broadly elliptic to orbicular and relatively small (4–21 × 4–17 mm) leaves, calyx of staminate flower shallowly plate-like, ovary rim conspicuously erose, and the urceolate capsule with a raised and lobed apical rim.

**Figure 2.**
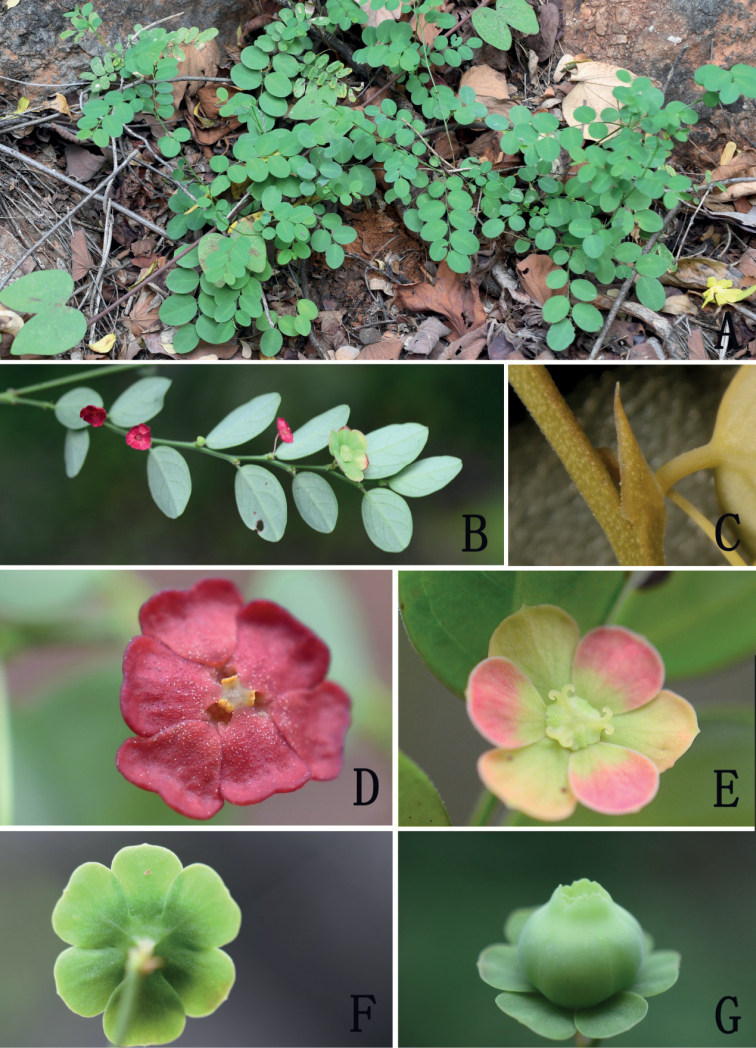
*Breyniahiemalis***A** habit **B** phyllanthoid branch showing pistillate flower and staminate flowers **C** stipule **D** staminate flower (apical view) **E** pistillate flower (apical view) **F** calyx in fruit (dorsal view) **G** fruit. Photographed by H. C. Wang from type locality in January 2022.

#### Description.

Dwarf shrubs or subshrubs, 10–20 (–30) cm tall, monoecious, glabrous throughout, with phyllanthoid branching. Main stems more or less procumbent to ascending, brown, with 4 shallow ribs, sometimes rooting at the lower nodes; branches green, deciduous, ascending, 3‒8 cm long. ***Cataphylls*** lanceolate, to 1 mm long, arranged spirally at the base of the plagiotropic branchlets. *Leaves* on ultimate branchlets distichous, simple; stipules triangular-lanceolate, usually auriculate basally, 1.5–2.0 mm long; ***petiole*** 1.2–1.6 × 0.3–0.5 mm; ***blade*** broadly elliptic to orbicular, rarely slightly ovate, papery, 4–21 × 4–17 mm, length/width ratio 1–1.5, base rounded to broadly cuneate, margin entire, flat, apex usually rounded, sometimes truncate, retuse, rarely mucronate, adaxially green, abaxially grey or slightly glaucous; venation pinnate, lateral veins 4 or 5 pairs, reticulate veins obscure. ***Inflorescences*** axillary, ***peduncles*** very short, ± 0.1 × 0.1 mm, with minute bracts, male or female flowers usually solitary, staminate flowers proximal, pistillate flowers usually distal. ***Staminate flowers*: *pedicel*** slender, ± 7 mm long; ***calyx*** shallowly plate-like, ± 4 mm in diam., red, 6-lobed; lobes biseriate, broadly obovate, slightly fleshy, 0.9–1.2 × 1.1–1.3 mm, apex obtuse or retuse, scales present; ***stamens*** 3, filaments connate, androphore ± 0.2 mm long, splitting horizontally, branches up to 0.5 mm long with anthers underneath, anthers ± 0.3 × 0.3 mm. ***Pistillate flowers*: *pedicel*** ± 3 mm long, thickening upwards; calyx ± 6 mm in diam., greenish, whitish yellow, or pinkish, lobes biseriate, obovate, subcoriaceous, outer lobes 2.5–3.0 × ± 2.5 mm, slightly longer and wider than the inner, inner lobes ± 2.5 × 2.1–2.5 mm, apex obtuse to truncate, shortly and abruptly acuminate; ***ovary*** obconical, 1.2–1.6 mm in diam., 3-locular, 2 ovules per locule, rim present at the apex, obviously erose; stigmas 3, spreading horizontally from top of ovary, apex split and recurved through ± 180°, sepals persistent and enlarged to ± 5 × 4 mm in fruit. ***Capsules*** urceolate, ± 4 × 5–6 mm, with a raised, lobed apical rim and persistent stigmas.

**Figure 3.**
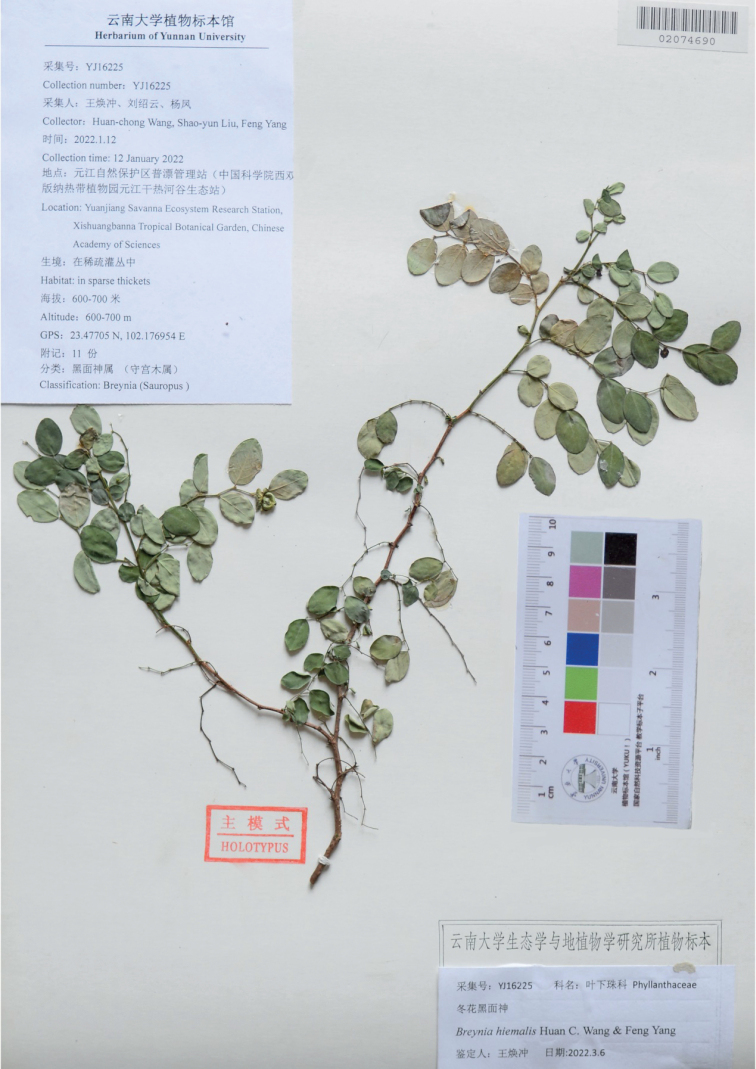
Holotype of *Breyniahiemalis* (YUKU-02074690).

#### Phenology.

Flowering from December to January, fruiting from January to February.

#### Etymology.

The epithet ‘‘*hiemalis*’’ is Latin for ‘‘belonging to winter’’, referring to the flowering period of this new species.

#### Distribution and habitat.

**Figure 4.**
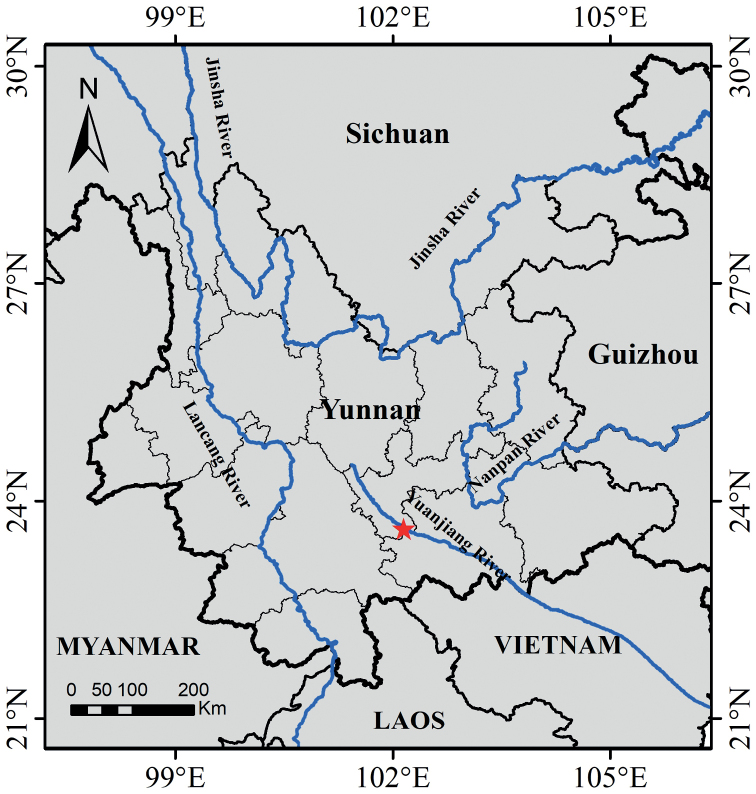
South-western China, showing the known distribution (red star) of *Breyniahiemalis*. The blue lines represent the rivers.

**Figure 5.**
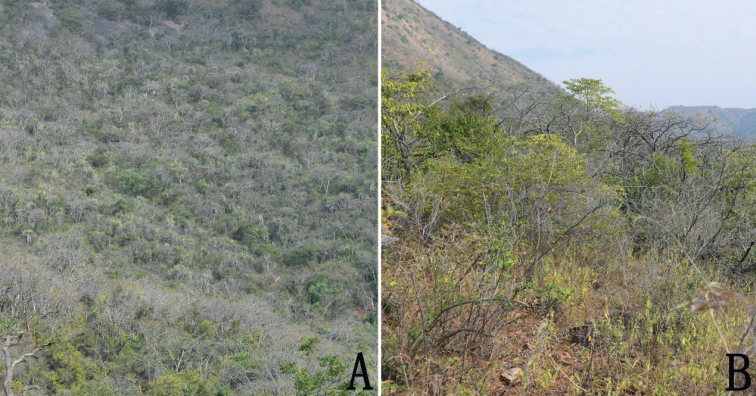
Habitat of *Breyniahiemalis***A** distant view of the type locality **B** nearby view.

*Breyniahiemalis* appears to be rare and is endemic to Yunnan, south-west China. It is known from only a single locality in the valley of the Yuanjiang River, which flows from Yunnan (south-west China) through northern Vietnam to the Gulf of Tonkin (Fig. 4). The climate in Yuanjiang valley is characterized by a long dry season (the dry season can be further divided into a cool dry season (November to February) and a hot dry season (March to April)), with an annual average temperature of 24 °C and a mean annual evaporation capacity of 2700–3800 mm, that is three to six times higher than the mean annual precipitation (600–800 mm), and with 80–90% of the precipitation concentrated in the wet season (from May to October) (Jin 2002; Shen et al. 2010; Zhou et al. 2017). *Breyniahiemalis* grows in savanna on a mountain slope (Fig. 5) at elevations of 500–700 m, together with *Lanneacoromandelica* (Houtt.) Merr. (Anacardiaceae), *Adina cordifolia* (Roxb.) Brandis (Rubiaceae), *Bauhiniabrachycarpa* Wall. ex Benth. (Fabaceae), *Tephrosiapurpurea* (L.) Pers. (Fabaceae), *Woodfordiafruticosa* (L.) Kurz (Lythraceae), *Waltheriaindica* L. (Malvaceae), *Jasminiummesnyi* Hance (Oleaceae), *Searsiapaniculata* (Wall. ex G. Don) Moffett (Anacardiaceae), *Heteropogoncontortus* (L.) P. Beauv. ex Roem. & Schult. (Poaceae), and others.

#### Additional specimens examined

**(*Paratypes*). China. Yunnan**: Yuanjiang County, Pupiao, 17 Dec. 2015, *H. C. Wang et al. YJ736* (YUKU).

#### Taxonomic notes.

**Figure 6.**
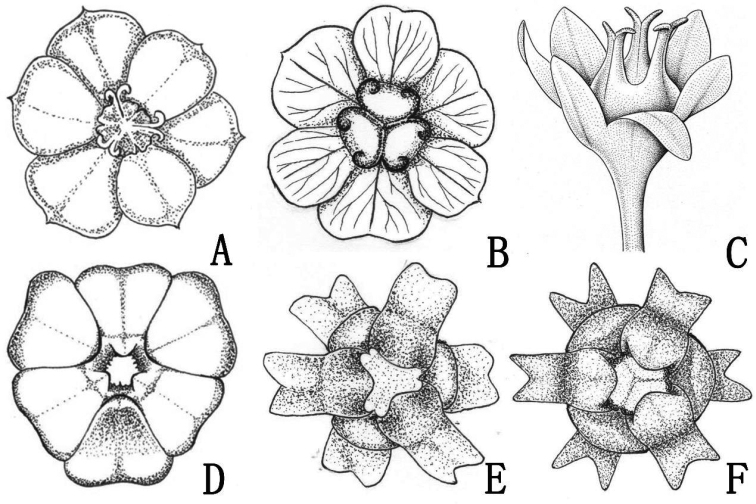
*Breyniahiemalis* (**A, D**), *B.compressa* (**B, E**), *B.granulosa* (**C**), *B.delavayi* (**F**). **A–C** pistillate flowers **D–F** staminate flowers.

According to Van Welzen et al. (2014) and Bouman et al. (2022), *B.hiemalis* should be assigned to sect. Cryptogynium because of its horizontal anthers (the androphore splits apically into three horizontal arms with the anthers hanging underneath) and ovary with a rim. Prior to the present study, only five species of sect. Cryptogynium were recorded in China, namely *B.compressa* (Müll. Arg.) Chakrab. & N. P. Balakr. (a member of the *B.quadrangularis* (Willd.) Chakrab. & N. P. Balakr. complex which was recognized as a distinct species by Chakrabarty and Balakrishnan (2015)), *B.delavayi* (Croizat) Welzen et Pruesapan, *B.pierrei* (Beille) Welzen et Pruesapan, *B.similis* (Craib) Welzen et Pruesapan and *B.tsiangii* (P. T. Li) Welzen et Pruesapan (Li and Gilbert 2008; Van Welzen et al. 2014; Chakrabarty and Balakrishnan 2015). *Breyniahiemalis* shows some resemblance to *B.compressa* and *B.delavayi* in its dwarf habit and axillary inflorescences. However, *B.hiemalis* can be distinguished from *B.compressa* by several characters, namely stems more or less procumbent to ascending (vs. erect or arching in *B.compressa*), calyx of the staminate flower shallowly plate-like (vs. star-shaped), lobes broadly obovate (vs. suborbicular or squarish), apex obtuse or retuse (vs. emarginate-truncate to deeply bilobulate) (Fig. 6: D, E), ovary rim erose (vs. retuse) (Fig. 6: A, B), capsule urceolate (vs. ovoid), with raised and lobed apical rim (vs. with low and smooth apical rim). Additionally, *B.hiemalis* flowers in winter (from December to January), whereas *B.compressa* flowers from summer to autumn (from April to October). *Breyniahiemalis* differs strikingly from *B.delavayi* in its obscure reticulate veins (vs. reticulate veins elevated on both surfaces) and calyx of the staminate flower shallowly plate-like (vs. star-shaped) (Fig. 6: D, F).

Of the species of *Breynia* found in south-east Asia, *B.hiemalis* is also morphologically similar to *B.granulosa* (Airy Shaw) Welzen & Pruesapan, from eastern Thailand. Nevertheless, *B.granulosa* differs from *B.hiemalis* in having obovate leaves (vs. broadly elliptic to orbicular, rarely slightly ovate in *B.hiemalis*), calyx of staminate flower campanulate (vs. shallowly plate-like), androphores ± 0.8 mm (vs. ± 0.2 mm) long, smaller pistillate flowers, usually 4–5 mm (vs. ± 6 mm) in diam., stigmas ascending (vs. horizontally spreading) (Fig. 6: A, C) and ovoid (vs. urceolate) capsules. *Breyniahiemalis* is also similar to *B.poilanei* (Beille) Welzen et Pruesapan from Vietnam, but it clearly differs from the latter by its more or less procumbent to ascending stems (vs. erect in *B.poilanei*), 0.1–0.2 (–0.3) m (vs. 1.5 m) tall, branches 3–8 cm (vs. 5–15 cm) long, stipules triangular-lanceolate (vs. triangular), 1.5–2.0 mm (vs. 0.5 mm) long, leaves broadly elliptic to orbicular, rarely slightly ovate (vs. ovate, rarely orbicular), capsules 4 mm (vs. up to 10 mm) wide. A key to distinguish the members of Breyniasect.Cryptogynium in China is given below.

### ﻿Key to Chinese species of Breyniasect.Cryptogynium

**Table:** 

1	Shrubs 1–3 m tall	**2**
–	Dwarf shrubs or subshrubs 0.1–1.0 m tall	**4**
2	Leaves ovate, 0.7–3.7 × 0.5–1.9 cm	***Breyniasimilis***
–	Leaves ovate to lanceolate, 6–10 × 3–4 cm	**3**
3	Leaves usually ovate, rarely ovate-lanceolate, papery, base rounded, apex caudate-acuminate	***B.tsiangii***
–	Leaves ovate-lanceolate to lanceolate, coriaceous, base cuneate, apex acuminate	***B.pierrei***
4	Calyx of staminate flower shallowly plate-like	***B.hiemalis***
–	Calyx of staminate flower star-shaped	**5**
5	Branches pubescent, leaves orbicular, base shallowly cordate, truncate or rounded, lateral veins robust, reticulate nerves prominent	***B.delavayi***
–	Branches glabrous; leaves ovate, elliptic or nearly rounded, base rounded or broadly cuneate, lateral veins slender, reticulate nerves obscure	***B.compressa***

## Supplementary Material

XML Treatment for Breynia hiemalis
